# A theoretical approach for estimating the effect of water-jet quenching on low-carbon steel beams

**DOI:** 10.1038/s41598-021-94819-9

**Published:** 2021-07-28

**Authors:** Bon Seung Koo

**Affiliations:** Technical Research Center, Hyundai Steel Company, Dangjin, Chungnam 31719 South Korea

**Keywords:** Engineering, Materials science, Mathematics and computing, Nanoscience and technology

## Abstract

Quenching is an efficient manufacturing technique to improve the strength of steel after hot rolling. The benefit of this application is to enhance the mechanical properties of steel products while reducing strengthening alloying elements, e.g., C, Mn, V, Nb, and N. Quenching and self-tempering (QST) especially for H-beams is a unique material strengthening process that adopts intensive surface cooling and self-tempering. A methodological difficulty in estimating the quenching effect has been a long-standing concern in the QST application. The purpose of this study was therefore to specify quenching parameters, quantify quenching, analyze the effect, and verify the credibility of the results. Transient quenching was simulated in ANSYS to analyze heat transfer and phase transformations due to quenching. An individual concept, e.g., heat exchange, cumulative quenching infiltration, or recalescence phenomena, was merged and interpreted newly for the quenching simulation. Computational results based on theoretical approaches were well consistent with empirical studies.

## Introduction

Heat treatments are used to enhance the mechanical properties of hot-rolled low-carbon steels. Quenching is one of the most common metal strengthening mechanisms related to the phase transformation of steel by rapid heat treatment. The benefit of quenching is to enhance the mechanical properties by grain refining while reducing expensive alloying elements. Quenching has therefore been used in many different fields of steel production.

Ajay et al. investigated the effect of oil quenching on the hardness of tool steels using the combination of different oils as quenchants^1^. Kiran and Leena introduced air jet impinging cooling on high carbon and chromium tool steels to achieve excellent hardness and moderate toughness^2^. Al-Khazraji et al. researched the use of polymers in place of water and oils for quenching cast aluminum alloys^3^. Fernandes and Prabhu examined the effect of different quenching media, e.g., water, oil, etc., on the heat transfer of high-carbon steels^4^. Ouchi described the technological advances in the manufacture of steel plates with thermo-controlled (TMC) and direct quenching (DQ) processes^5^. TMC is a generalized definition of many applicable rolling processes which can be sorted out by proposed temperature control. DQ is a manufacturing process that rapidly cools steels from the austenitization temperature to room temperature with an extremely high cooling rate. DQ has been developed as one of the TMC processes for steels with good toughness and weldability due to advances in accelerated cooling. Accelerated cooling is a quenching process that spurts pressurized water onto a hot surface to obtain a fine microstructure corresponding to higher mechanical properties. The beam quenching process consists of quenching and self-tempering (QST) rather than DQ preferred in steel plates. The QST process was developed by ARBED, CRM, and British Steel^6^, and it was also considered an enhanced heat treatment technique similar to the TMC process. Quenching in QST is an intensive surface cooling technique that uses high-pressure water as a coolant for only a few seconds. Self-tempering is a process of surface reheating through heat transferred from the core which is not yet cooled due to quick quenching.

The advantage of quenching is more pronounced if the process is under control. MacKenzie used computational fluid dynamics (CFD) for uniform oil quenching of automotive gears in a quenching vessel^7^. Chaudhari et al. investigated the effect of quenching parameters on the hardenability of tool steels using a CFD approach^8^. Similarly, Passarella et al. used a commercial tester to measure the temperature of the oil-quenched probe and calculated a heat transfer coefficient (HTC) in reverse order^9^. Ma et al. developed a lab-scale heating, cooling, and data acquisition system to calculate an effective HTC in mineral oil quenching^10^. Hasan et al. measured the HTC of steels in water quenching based on temperature and cooling rate^11^. Agboola et al. applied a finite difference method to calculate the temperature change of medium carbon steels that were released into a water container from a certain height^12^. Recently, Bouissa et al. utilized a finite element computation to estimate an HTC for quenching of large steel blocks^13^.

Despite the various studies on quenching, the understanding of QST has been very limited due to the complexity of the quantification. Most of the preceding quench studies used feedback to modify and update an HTC based on the measured temperature. QST is however an online manufacturing process whose products are constantly influenced by quenching parameters. The trial-and-error approach in real manufacturing was not desired due to cost and productivity losses. A basic guideline was essential to control the quench parameters instantaneously and to predict the potential metallurgical behavior due to quenching. A quantitative measure of quenching was therefore highly required to evaluate the effect in advance. A theoretical approach to understanding the quenching effect was the primary objective of this research, and experimental data were analyzed to verify the suitability of the proposed methods.

## Materials and procedures

A research material was SHN355 hot-rolled H-shaped steel beams. The dimensions of the beams used were given in Table 1 according to the cross-sectional geometries described in the Korean Standard. $H$ [mm] and $B$ [mm] stand for the length of the web and flange respectively, and ${t}_{2}$ [mm] means the thickness of the beam flange. The material requires a minimum yield strength (YS) of 355 MPa, a tensile strength (TS) of up to 610 MPa, and a YS/TS ratio of 85% as in KS D 3866^14^. Table 2 is the chemical compositions of SHN355, and Table 3 is the corresponding mechanical properties.

**Table 1:**
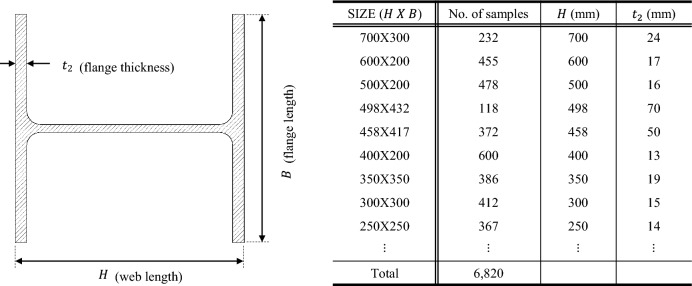
A group of H-beams used for quenching analysis.

**Table 2:** Chemistry requirements of SHN355 in KS D 3866 standard.

KS D 3866	max. wt%											
	C	Si	Mn	P	S	Cu	Ni	Cr	Mo	V	Nb	Mn/S
SHN355	0.20	0.40	0.5 ~ 1.5	0.035	0.030	0.60	0.45	0.35	0.15	0.11	0.05	20↑

**Table 3:** Mechanical characteristics of SHN355 in KS D 3866 standard.

KS D 3866	YS [N/mm^2^]	TS [N/mm^2^]	YS/TS [%]	Elongation [%]		Impact toughness [J]
				${t}_{2}\le 40$ mm	${t}_{2}>40$ mm	at 0 ℃
SHN355	355~475	490~610	85	21	23	27

**Figure 1:**
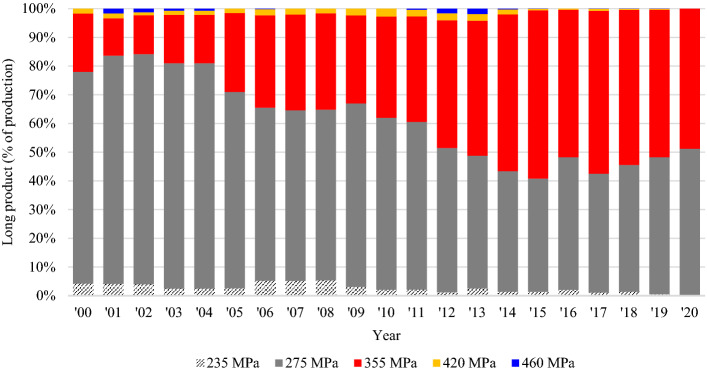
Analysis of long product sales trends.

In the building industry, low-carbon steels are economical materials for their long-term sustainability. The strength of steel is an important decision-making factor in building construction and applications. Steel grades in YS classes up to 275 MPa were mostly used in the 1970s for general construction^15^. The use of steel beams with a minimum YS of 355 MPa has been progressively increased over the last decades. A 20-year sales trend for long products, e.g., H-beams, angles, and channels, was analyzed to estimate a current preferred steel grade in the construction market. Figure 1 shows the trend in the annual production of long products at Hyundai Steel Works in Incheon, South Korea. The sales rate for 355-MPa grade steel has shown an upward trend during the period. As a result, SHN355 steel beams were selected for this quenching study based on the current steel preference and its relevance to the quenching process.

**Figure 2:**
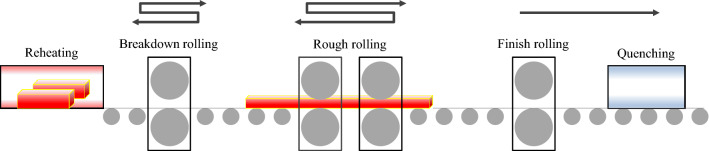
Schematic diagram of the beam manufacturing process.

A hot-rolling and quenching process was conducted in the Incheon works of Hyundai Steel. H-beam rolling was classified into three separate processes, e.g., reheating, rolling (break down, roughing, and finishing), and quenching. A workpiece called a beam-blank extracted from a reheating furnace is transported to a break-down mill forming a rough “H” shape. It was then transferred to a universal roughing-edging mill to form a required "H" shape. The key process factors in roughing rolling were a start rolling temperature (SRT) and a finish rolling temperature (FRT) to acquire the desired microstructure. Rolling was implemented to achieve the decent straightness of H-beams with a few parameter controls. The product dimension and tolerance requirements, e.g., straightness and squareness, were finally obtained in a finishing rolling process. The rolled beams were then surface-hardened by a rapid phase transition in quenching. The overall H-beam manufacturing process is outlined in Fig. 2.

**Figure 3:**
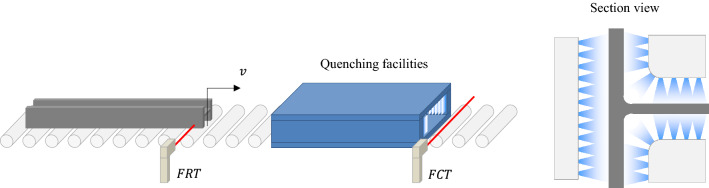
Quenching process for hot-rolled H-beams.

Quenching was conducted with appropriate control of input parameters for uniform heat treatment of materials. An important quenching parameter, a finish cooling temperature (FCT), was determined to increase or decrease the effect of quenching. A travel speed and quenching coolant flow rate were controlled to achieve the required FCT. The simplified quenching process is described in Fig. 3. The rate of coolant flow was given as a variable if a beam travel speed was constant, and vice versa.

**Figure 4:**
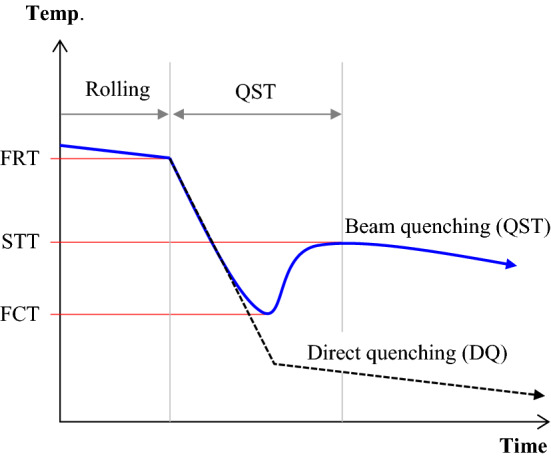
Typical QST cooling scheme versus DQ.

Figure 4 depicts the QST cooling pattern. The QST process was primarily designed for beam-like sections, and the main purpose of the QST application is as follows: 1) to increase in tensile properties, 2) to enhance toughness at low temperatures, 3) to improve high weldability by a low carbon equivalent, and 4) to lower manufacturing costs by the reduced contents of alloying elements.

## Theoretical approach

### Inverse estimation of heat transfer coefficient in quenching

Hot-rolled steel beams are quenched when a high-pressure water-jet impinges on the surface. The quench severity is relevant to the rate of heat extraction, and it refers to how quickly heat can be drawn out of the substance. The effect of quenching therefore depends on the water flow rate and the exposure time of the material to the water-jet. A theoretical approach to heat transfer is based on a heat exchange relationship between hot and cold media. The beam was considered a heat source, and thermal energy was transferred to water through the thermal contact between the beam and the water. The heat input shall be balanced with the amount of heat output according to the principle of energy conservation. Heat exchange was assumed to involve the interactions of thermal energy which occurred across a liquid–solid boundary. This hypothesis included primary thermal reactions, e.g., convection and vaporization. The expression of the thermal energy balance is described in Eq. (1).

1 $${q}_{\text{convection}}={q}_{\text{water}}+{q}_{\text{vaporation}}$$

**Figure 5:**
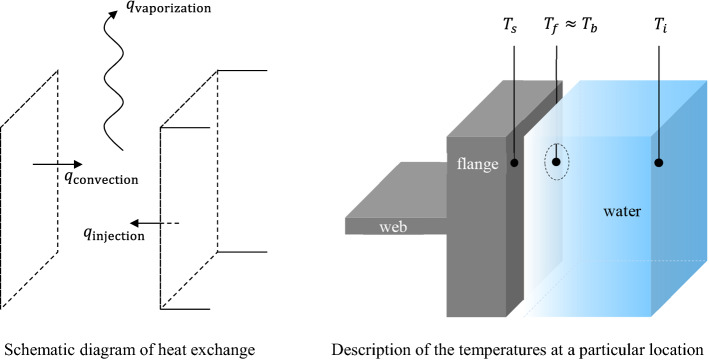
Heat exchange between hot and cold media.

Figure 5 shows the thermal boundary conditions, e.g., the distribution of temperatures and heat flux, where the heat exchange occurs. The beam had higher thermal energy than the cold water; therefore, the heat was transferred from the beam surface to the water by convection. The expression for convective cooling is expressed in Eq. (2).

2 $${q}_{\text{conv}}=h\left({T}_{s}-{T}_{i}\right){A}_{s}$$

$h$ is the heat transfer coefficient caused by forced convective quenching, and ${A}_{s}$ is the infinitesimal unit area of the solid–liquid contact surface. ${T}_{s}$ and ${T}_{i}$ are a beam flange temperature at the surface and an initial water temperature measured before quenching. The water temperature rises from room temperature (initial temperature, ${T}_{i}$) to the boiling point (finial temperature, ${T}_{f}$), and some water vaporizes instantly due to a huge amount of heat energy supplied by the hot flange. To define the relationship between the temperature rise and the amount of heat delivered, the heat transferred from one system to another is given by the movement of fluids as specified in Eq. (3). This involves achieved thermal energy and liquid–vapor transition energy.

3 $$q={c}_{w}\dot{m}\Delta T={\rho }_{w}\left[{c}_{w}\left({T}_{f}-{T}_{i}\right)+\Delta {H}_{v}\right]\dot{Q}$$

${c}_{w}$, ${\rho }_{w}$, and $\dot{m}$ are the specific heat, the density, and the mass flow rate of water sprayed at high pressure. $\Delta {H}_{v}$ is the latent heat of water vaporization, and $\dot{Q}$ [m^3^/h] is the volumetric flow rate derived from $\dot{m}$ [kg/s] which is applied to the unit area of thermal contact. The meaning of the symbols and the thermo-physical properties required are given in Table 4. The amount of heat released must be balanced with the heat transferred throughout the system. The thermal energy of Eq. (2) is assumed to be the same as the heat exchanged of Eq. (3) based on the principle of energy conservation. Therefore, the environment variable $h$ can be obtained using the law of energy conservation once the volumetric flow rate is correctly converted. A precise approximation of $\dot{Q}$ is addressed in the following section considering a moving workpiece during quenching.

**Table 4:** Thermo-physical properties for calculating transferred heat.

Name	Symbol	Unit	Value
Quenching heat transfer coefficient	*h*	W/m^2^·K	(to be calculated)
Surface temperature (beam)	${T}_{s}$	°C	(to be calculated)
Final temperature (water)	${T}_{f}$	°C	≈ 100
Initial temperature (water)	${T}_{i}$	°C	≈ 33
Boiling point (water)	${T}_{b}$	°C	100
Heat of vaporization (water)	$\Delta {H}_{v}$	kJ/kg	2257
Specific heat (water)	${c}_{w}$	J/kg·°C	4179
Density (water)	${\rho }_{w}$	kg/m^3^	997

### Numerical integration of cumulative quenching infiltration

A quench severity is dependent on the application time and flow rate. A time-dependent approach is indispensable to quantify the effect of a water-jet because quenching occurs while the target material is moving. Assuming that the accumulated amount is a function of time and position, an infinitesimal amount should be set for a specific position in time. Integers $i$ and $j$ are therefore used as the indices discretizing the accumulation in the given time-position domain. ${\lambda }_{j}$ is the accumulation at a specific position $j$, and ${\varphi }_{i}$ is defined as an infinitesimal amount of the water-jet at a given time $i$ as of Eq. (4).

4 $${\lambda }_{j}={\left(\sum {\varphi }_{i}\right)}_{j}$$

The unit flow rate $\dot{Q}$ in Eq. (3) should be redefined to reflect the cumulative water-jet effect, thus a numerical summation has been proposed as shown in Eq. (5).

5 $$\sum {\varphi }_{i}=\underset{n\to \infty }{\text{lim}}\sum_{i=1}^{n}{\varphi }_{i}=\underset{n\to \infty }{\text{lim}}\sum_{i=1}^{n}\frac{\dot{{Q}_{i}}}{n}$$

A constant $n$ is considered an extremely large number to discretize the water-jet accumulation and has a relation of $L=n \Delta \xi$. $L$ [m] is the overall length of the quenching facilities. $\Delta \xi$ stands for a small distance-step size where $\xi$ indicates a specific position within the beam exposed to the water-jet. Assuming the beam is drawn with a speed of $v$ [m/s], the relation of $\Delta \xi =v\Delta t$ becomes readily applicable. $\Delta \xi$ is a small distance traveled at a speed of $v$ during the time interval of $\Delta t$. The travel speed $v$ is usually given as a process parameter that has an important role in quench hardening and cooling efficiency.

6 $$\sum {\varphi }_{i}=\underset{\Delta t\to 0}{\text{lim}}\sum_{i=1}^{n}\frac{\dot{{Q}_{i}}}{L}v\Delta t=\frac{v}{L}{\int }_{0}^{t}\dot{{Q}_{i}}dt$$

${\lambda }_{j}$ represents the cumulative effect linked to a specific position and thus corresponds to all the values obtained at each time step. The elapsed time increases from ${t}_{1}$ to ${t}_{i}$, and the index $i$ denotes an arbitrary time step assumed to be in the range of $1\le i\le j$. Finally, the sum of ${\lambda }_{j}$ results in the cumulative effect of the overall water-jet which included all heat exchanges across the beam. The position index $j$ is placed in the range of $1\le j\le n$ because the beam is supposed to move sequentially to the ${n}$th discretized step-distance.

7 $$\sum {\lambda }_{j}={\lambda }_{\text{max}}\leftrightarrow \dot{Q}$$

**Figure 6:**
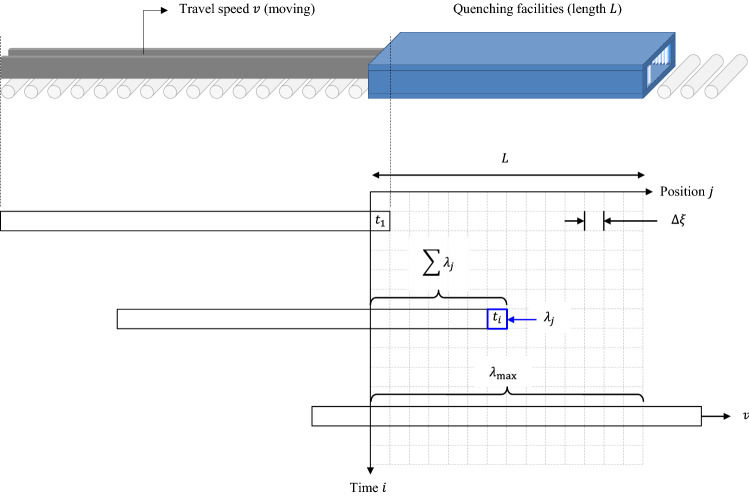
Numerical integration of quenching effect.

**Figure 7:**
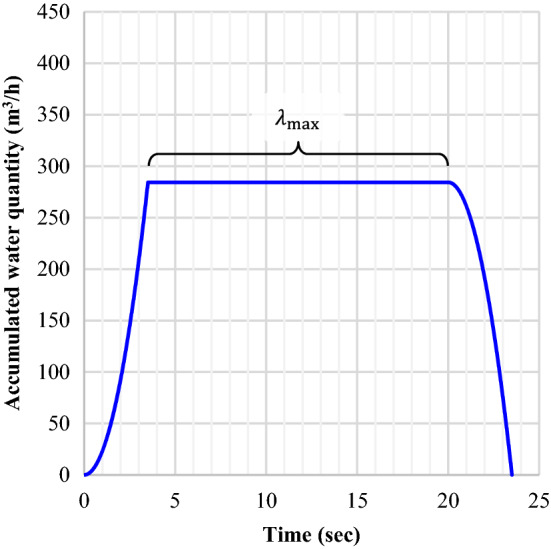
Accumulated profile of the quench.

The beam could be completely soaked within a few steps after quenching begins (as shown in Fig. 6). It implies that the cumulative effect would be uniform throughout the beam without the variations in the water-jet accumulation. The particular value of this stage is defined as ${\lambda }_{\text{max}}$ [m^3^/h] which contributes to a consistently high quenching effect. $\dot{Q}$ in Eq. (3) is only described in terms of the supplied water, but the real effect of heat treatment is derived from an environmental impact delivered to the beam. It explains that the amount of water involved in heat exchange is more practical than the amount of water released (as in Eq. (7)). Figure 7 shows the result of this calculation.

### Mathematical estimations of recalescence

A phase transformation is a multi-physical process in which a phase of a substance turns into a new or different phase. The change in thermodynamic parameters promotes phase transformation and usually has a substantial effect on the change in thermophysical properties, e.g., density, specific heat, thermal expansion coefficient, etc. Temperature is one of the thermodynamic parameters which indicate the physical characteristics of steels by the amount of heat released or absorbed.

When a hot-rolled beam cools, its temperature would eventually fall below Ar3 during the quenching process. Austenite begins to transform to ferrite, and the austenite-to-ferrite transition is promoted by various cooling rates. As the carbon content of low-carbon steels is limited up to 0.2%, most structural steels are hypoeutectoids. When further cooling occurs below Ar1, untransformed austenite becomes pearlite in slow cooling. However, quenching is extremely rapid; carbon is typically trapped in an iron lattice and results in new structures, e.g., bainite and martensite, in the alpha phase.

**Figure 8:**
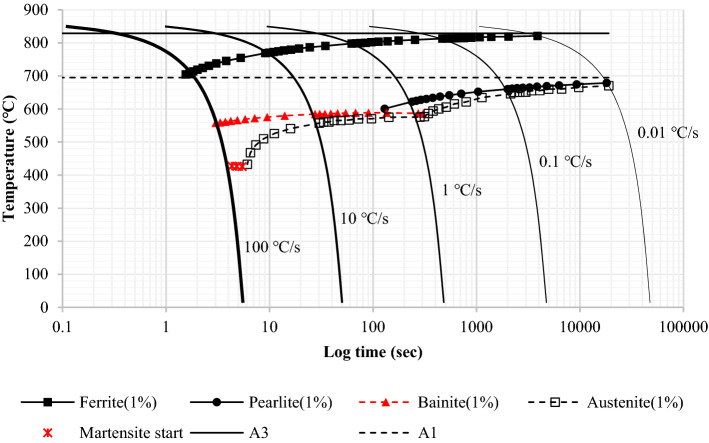
CCT diagram of Nb-V-Al micro-alloyed low carbon steels (JMatPro).

Recalescence is an event of heat release due to phase transformation. Austenite transforms to ferrite, pearlite, bainite, and martensite respectively depending on the cooling rate as shown in Fig. 8. Austenite is supposed to change gradually or rapidly depending on the cooling profile, and the fraction of the transformed phase varies according to the cooling rate. Therefore, a new transformed phase releases a different amount of latent heat based on the thermodynamic conditions.

The fraction of phase change is determined by the rate of cooling and temperature profile along the cooling path. Each phase transformed from austenite has a specific density and latent heat of fusion. As phase transformation releases heat energy in cooling, heat generation $q$ [W/m^3^] due to the phase change is a product function of the phase fraction, density, and specific latent heat as shown in Eq. (8).

8 $$q\left({T}_{i,j}\right)=\Delta f\left({T}_{i,j}\right)\rho \left({T}_{i,j}\right)L\left({T}_{i,j}\right)/\Delta t$$

As described above, $i$ and $j$ are the indices representing a specific time and position of the beam. $L$ [J/kg] is the latent heat of transformation, and $\Delta f$ corresponds to the volume fraction which has been phase-transformed in the given temperature. $\Delta t$ is given as 0.01 s to convert energy into power.

Phase transformation in quenching was assumed to occur continuously from 0 to 99%. Leblond et al. proposed a model for the transformed volume fraction based on Scheil’s additivity rule^16^, and Simir and Gur mathematically developed the additivity rule to apply in thermo-physical processing^17^. Phase transition in continuous cooling and the volume fraction of each phase was predicted by reviewing the additivity studies. Latent heat was released proportional to the change in phase volume fraction. Guo et al. studied the overall phase transformation kinetics based on Avrami theory^18^, and their models have been modified to define a volume fraction of the transformed phase as shown in Eq. (9).

9 $$\Delta {f}_{i}=1-\frac{{f}_{i}-{f}_{i-1}}{{f}_{tot}}$$

10 $$\Delta f=1-\sum \Delta {f}_{n}$$

${f}_{i}$ stands for a volume fraction of transformed phase at a time step $i$*,* ${f}_{tot}$ is the total volume fraction, and $\Delta {f}_{i}$ denotes a time-dependent instantaneous volume fraction during the phase change. Equation (10) implies an infinitesimal volume fraction of $\Delta f$ which corresponds to a specific phase, and $\sum \Delta {f}_{n}$ implies the rest of the total.

**Figure 9:**
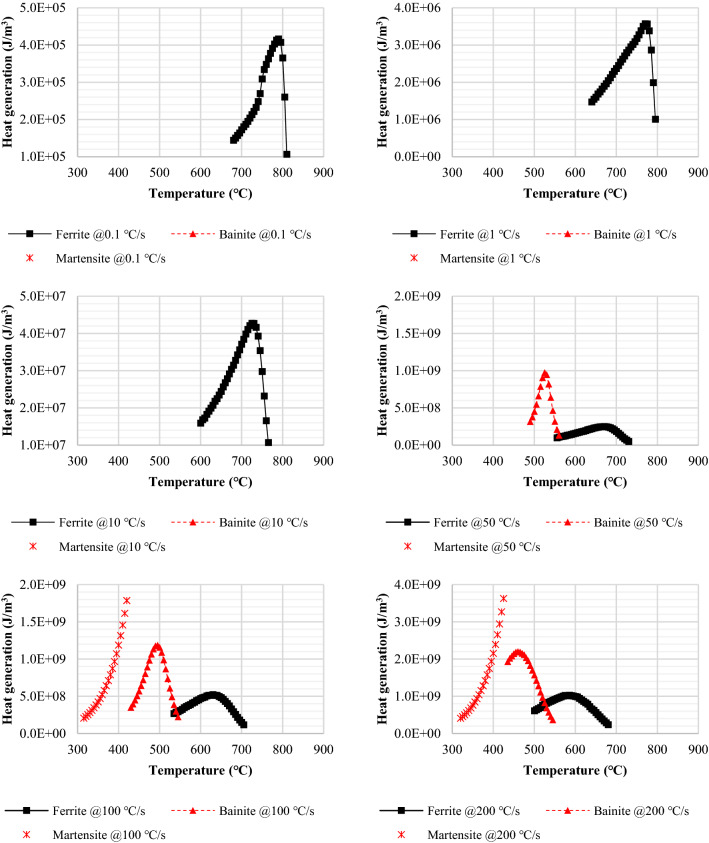
Heat generation calculated at various cooling rates.

In many simulation experiments, the latent heat per unit volume (called heat density) below 1 × 10^6^ [J/m^3^] was considered negligible (not affecting any perceptible rise in temperature) when the quenched surface cooling rate was in the range of 100 ~ 200 °C/s. As QST is usually completed in less than 10 s, a heat density over 1 × 10^7^ [J/m^3^] seemed more adequate to make a significant change in temperature. Figure 9 is the profile of heat generation calculated at various cooling rates. The heat generated is formulated according to temperature and cooling rate. And it is plugged into the energy conservation principle to update the temperature field in the transient quenching simulation.

11 $$\frac{\partial }{\partial x}\left(k\frac{\partial T}{\partial x}\right)+\frac{\partial }{\partial y}\left(k\frac{\partial T}{\partial y}\right)+\frac{\partial }{\partial z}\left(k\frac{\partial T}{\partial z}\right)+q=\rho {c}_{p}\frac{\partial T}{\partial t}$$

$k$·[W/m·K] is the thermal conductivity of the beam and assumed constant for fast nonlinear analysis because the rate of heat transfer by heat generation is much higher than the conductive heat transfer. ${c}_{p}$ is the specific heat of the beam and also assumed constant over temperature due to the already applied phase transformation heat as in Eq. (11). Table 5 shows the thermo-physical properties of the beam used in the computation. The density is changed during the cooling cycle, so the non-linearity of the density was applied to compute the recalescence effect (as of Eq. (8)). However, the density was treated constant in the governing equation (as of Eq. (11)) because there was no dramatic change in the magnitude and no need to make the density nonlinear again.

**Table 5:** Thermo-physical properties of a beam.

Name	Symbol	Unit	Value
Thermal conductivity (beam)	$k$	W/m·K	27
Density (beam)	$\rho$	kg/m^3^	7,745
Specific heat (beam)	${c}_{p}$	J/kg·°C	800

## Manufacturing data analysis

A large number of manufacturing data were analyzed to quantify the effect of quenching. The data were categorized into separate quenching groups according to the size of H-beams. The H-beam dimensions in Table 1 have been used for quenching analysis. The selected models represent a range of sizes corresponding to wide and narrow H-beams. $H$ (the width of the web) varies from 200 to 700 mm, and $B$ (the height of the flange) varies between 200 and 400 mm approximately according to the specified geometries. ${t}_{2}$ is the thickness of the flange that varies between 11 and 70 mm. In general, the quenching effect is proportional to the coolant flow rate and inversely related to the beam travel speed due to the quenching retention time. More intensive quenching can result in a greater temperature variation between FRT and FCT $(\Delta T=FRT-FCT)$, and the thinner H-beams are more sensitive to quenching. These process attributes could be presented as a correlation, as shown below.

12 $$f\left( {\dot{Q},v^{{ - 1}} } \right) \propto \Delta T,t_{2} \Rightarrow \frac{{\dot{Q}}}{v} \propto \Delta T \cdot t_{2}$$

**Figure 10:**
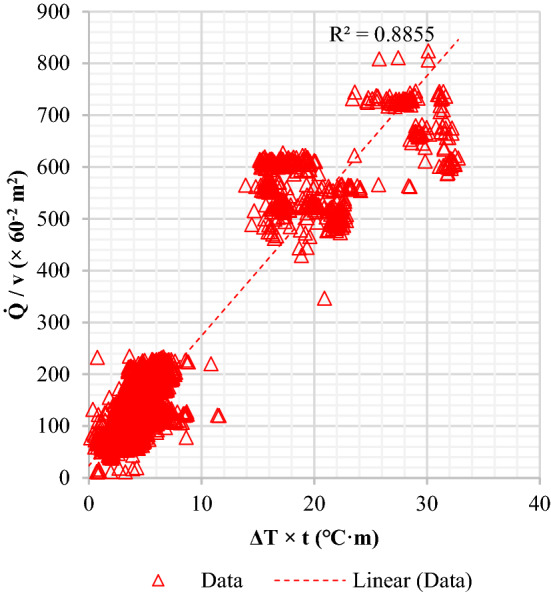
Plot of quenching characteristics using combined variables.

The massive manufacturing data were analyzed by linear regression models using two combinatorial variables, $\dot{Q}/v$ and $\Delta T \cdot {t}_{2}$, which were set to independent and dependent variables respectively. The measured R-squared value of the linear regression plot became 0.88 as shown in Fig. 10. The R-squared showed how precisely the data was approximated to the regression line. As a result of the least-squares approach, the quenching effect could be simply defined with a few quenching parameters and resultants.

## Results and discussion

### Suitability of simulation results

A series of quenching simulations were conducted by varying the beam travel speed and the coolant flow rate to evaluate the effect of quenching. The efficacy was estimated by measuring the temperature difference before and after quenching. Heat transfer during quenching was calculated using a finite element (FE) program ANSYS. The calculation was performed on a 2D symmetry model using the same field quenching operating parameters. A half symmetry of the H-beams was modeled to save computation time while avoiding repetitive geometric patterns. In the FE analysis, elements of 2 mm × 2 mm (rectangular mesh) were consistently applied throughout the overall half-beam model. The number of elements for a single calculation was increased up to 10,000 according to the size of the beam. The 2D thermal analysis included both radiation and convection considering heat dissipation to the surrounding before and after quenching. The quench simulation used an HTC that was derived from the predefined numerical flow rate ${\lambda }_{\text{max}}$ based on the cumulative infiltration calculation. The analysis covered the most frequently mass-produced H-beams over the years. The quenching parameters in Table 6 were also used to reproduce the empirical tests.

**Table 6:** Beam dimensions and quenching parameters.

SIZE ($H\times B$)	${t}_{2}$(mm)	$\dot{Q}/v$(× 60^–2^ m^2^)
700 × 300	24	203
600 × 200	17	84
500 × 200	16	84
498 × 432	70	730
458 × 417	50	491
400 × 200	13	67
350 × 350	19	210
300 × 300	15	131
250 × 250	14	108

**Figure 11:**
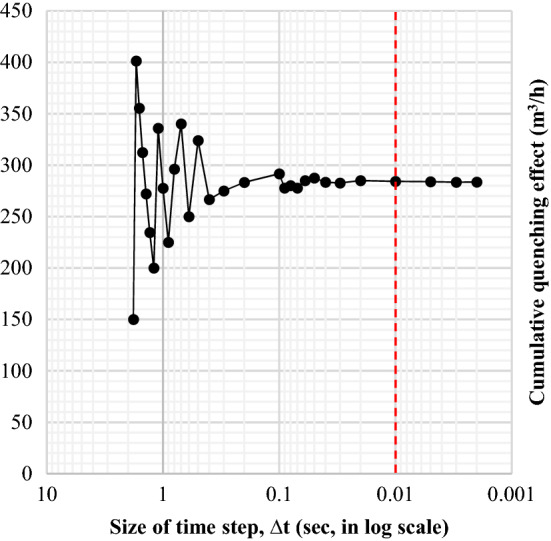
Improvement of the FEM convergence by reducing the time step size.

A theory of cumulative quenching infiltration was the hypothesis to quantify direct quenching on materials. The stability of the calculation was enhanced by reducing the time-step size, and the credibility of the theoretical approach was considerably improved. Figure 11 shows the significantly increased stability of the cumulative quenching when the time step scales have been minimized in seconds to milliseconds. As a result of numerical calculations, a 0.01-s interval was considered appropriate for reliable results.

**Figure 12:**
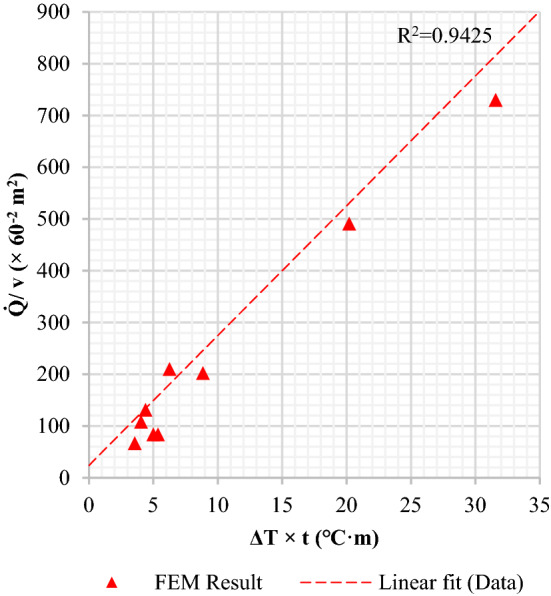
Comparison of FEM suitability with production results adjusted to the regression line.

The reliability of FE computation results was evaluated using the same empirical regression line described in the earlier section. The calculation result was described in Fig. 12 using the combinatorial quenching variables. The red dashed line is the regression line fitted to the 6,820 empirical data points. The red triangle symbols indicate the results of the FE calculation. The graph shows that the FE results have an R-squared value of 0.94, which is higher than the empirical value of the same regression line. The comparison demonstrates that the quenching effect can be more predictable using cumulative quenching infiltration.

### Quenching simulation and recalescence effect

**Figure 13:**
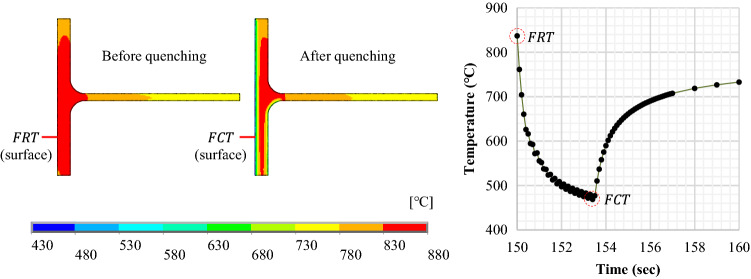
Temperature change during QST with regards to FRT and FCT.

One of the biggest size beams was chosen to discuss the simulation results. Figure 13 shows the computation result of an H700X300 beam. As QST implemented intensive surface cooling, a surface subjected to the pressurized water-jet cooled off rapidly in a few seconds. And then the temperature of the quenched surface was restored back to a steady-state over time by self-tempering.

**Figure 14:**
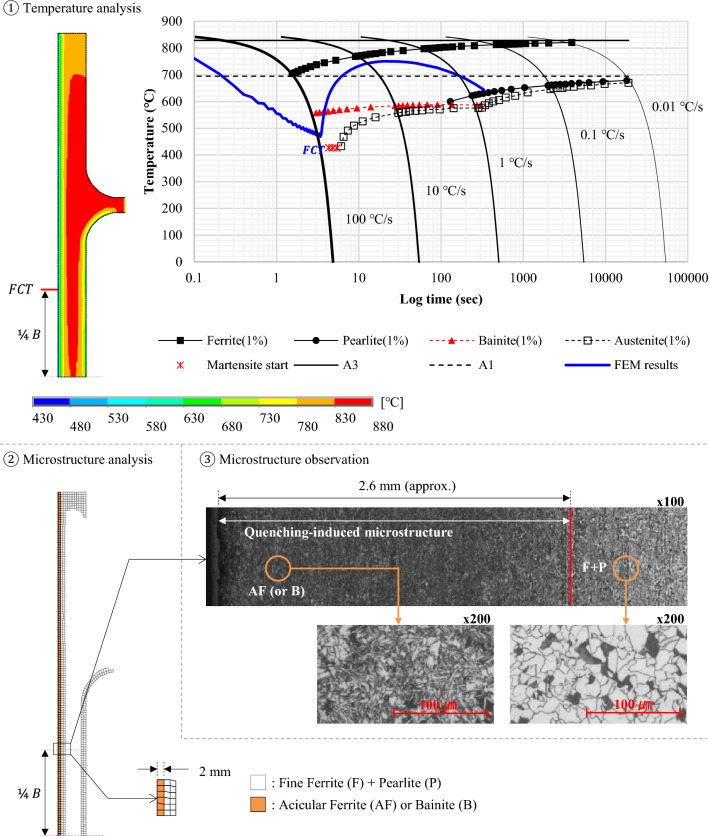
Comparison of microstructure observations of computation results.

The temperature and cooling rate calculated during the thermal analysis were used to define a transformed phase of the material. Figure 14 shows the FE results compared with microstructure observation. The temperature profile at the lower ¼ of the flange surface (same location as the process in production) was calculated using ANSYS, and the cooling curve superimposed on the CCT diagram was given to estimate the quench-induced phase transformation.

The microstructure was then observed using an optical microscope and an image analyzer after etching. The microstructure of acicular ferrite (AF, needle-shaped grains), which was often referred to as a low-temperature phase transition structure, was obtained on the very surface. A mixture of ferritic-pearlitic microstructures was found slightly off the surface. A much finer microstructure could form on the surface than the inside as the QST was a surface cooling process. The calculation also showed that the H-beam surface exhibited an AF (or bainite, B) microstructure due to an extreme cooling rate. Zhao et al. described that the fraction of AF laths was increased when the cooling rate was increased to 35 °C/s^19^. A cooling rate above 35 °C/s was therefore assumed to promote the formation of AF. Quenching was favorable for the grain refining of low-carbon steels, and its strong application could be more effective for further austenite-to-AF (or bainite) or -to-martensite phase transformation. The prediction of microstructure evolution seemed well suited to presume the metallurgical effects of quenching.

The calculation results were also analyzed to verify the effect of latent heat during the phase change. Recalescence can increase the temperature by releasing latent heat as quenching promotes phase transformation. In Fig. 14, the FCT should be above the martensite-start temperature (Ms) as in the microstructure observation which showed acicular ferrite (or bainite) at the surface. The first simulation however resulted in a much lower FCT than expected. This temperature inconsistency often occurs in the quenching simulation when compared to empirical findings. An exothermic reaction during quenching was therefore assumed to be one of the factors that produced uncertainty in the temperature estimate.

**Figure 15:**
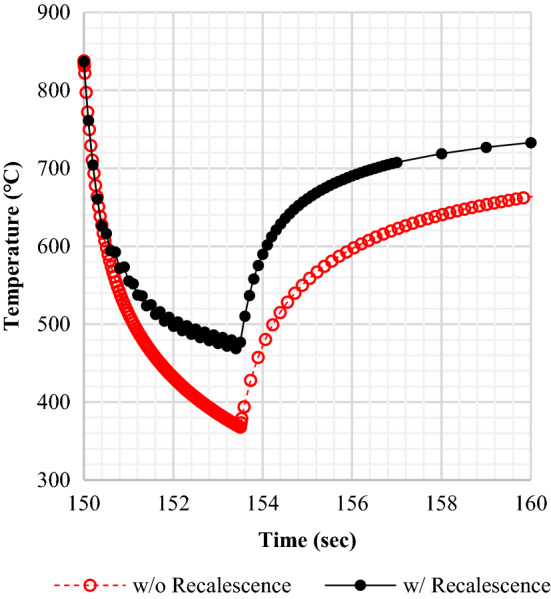
Effect of recalescence on the FCT difference.

Figure 15 is an assessment of how recalescence affects temperature change during the QST process. A solid black line represents the result including recalescence. The FCT estimate falls several degrees below 500 °C but higher than the Ms temperature of about 415 °C. On the other hand, a dashed red line is a result that occurred without recalescence. The computation led to an abnormal temperature drop below the target. This phenomenon proved that the calculation of temperature with recalescence was in good agreement with the actual data. As a result, the estimation of FCT would become more reliable even if latent heat was generated by quenching.

## Conclusions

The quantification of beam quenching has been a theoretically unresearched area for a long time. The theoretical estimate of the quenching effect includes many uncertainties, and it has been mostly dependent on experiments and experimental correlation. The fundamental objective of this study is to use all available theoretical resources to quantify the quench and quench effect. There are still many practices required to improve the precision of mathematical implementations, but a major achievement of this study is that quenching becomes more theoretically predictable. According to this research work, the results lead to the following conclusions:

- The fundamentals of heat exchange in quenching are the heat transfer from the heated beam to cold water.
- The numerical integration of water-jet injection is indispensable to quantify the effect of quenching involved in heat exchange.
- The quenching effect can be determined by several parameters, e.g., the amount of water released, the speed of a beam passing through the quenching facilities, a beam thickness, and the temperature drop due to quenching.
- Recalescence is a unique metallurgical phenomenon that is observed during phase change and releases a substantial amount of heat if quenched at a high cooling rate.
- The effect of phase transformation is more clearly observed by using FE analysis, while the recalescence phenomenon during real QST is less noticeable due to instantaneous heat recovery.

## Abbreviations

CCT: Continuous cooling transformation
CFD: Computational fluid mechanics
CR: Controlled rolling
DQ: Direct quenching
FCT: Finish cooling temperature (℃)
FRT: Finish rolling temperature (℃)
HTC: Heat transfer coefficient
QST: Quenching and self-tempering
SRT: Start rolling temperature (℃)
STT: Self-tempering temperature (℃)
TMC: Thermo-mechanically controlled
T_NR_: Non-recrystallization temperature (℃)
TS: Tensile strength (MPa)
YS: Yield strength (MPa)
