# Author Correction: A theoretical approach for estimating the effect of water-jet quenching on low-carbon steel beams

**DOI:** 10.1038/s41598-021-03591-3

**Published:** 2021-12-15

**Authors:** Bon Seung Koo

**Affiliations:** Technical Research Center, Hyundai Steel Company, Dangjin, Chungnam 31719 South Korea

Correction to: *Scientific Reports* 10.1038/s41598-021-94819-9, published online 28 July 2021

The original version of this article contained an error in Table 3, where the values for “YS [N/mm^2^]”, TS “[N/mm^2^]”, and “Impact toughness [J] at 0 °C” were incorrect. The incorrect and correct values appear below.

Incorrect:

**Table:** 

KS D 3866	YS [N/mm^2^]	TS [N/mm^2^]	YS/TS [%]	Elongation [%]		Impact toughness [J]
				*t*2 ≤ 40 mm	*t*2 > 40 mm	at 0 °C
SHN355	460	540/720	85	21	23	20

Correct:

**Table:** 

KS D 3866	YS [N/mm^2^]	TS [N/mm^2^]	YS/TS [%]	Elongation [%]		Impact toughness [J]
				*t*2 ≤ 40 mm	*t*2 > 40 mm	at 0 °C
SHN355	355~475	490~610	85	21	23	27

The original Article has been corrected.

